# Report of Surgical Cases, Treated at St. James’ Hospital, Chicago, during the Months of July and August

**Published:** 1857-10

**Authors:** Geo. K. Amerman

**Affiliations:** One of the Attending Surgeons


THE NORTH-WESTERN

MEDICAL AND SURGICAL JOURNAL.

(NEW SERIES.)

VOL. VI. OCTOBER, 1857. NO. 10.

### ORIGINAL COMMUNICATIONS.

ARTICLE I.—*Report of Surgical Cases, treated at St. James' Hospital, Chicago, during the months of July and August.* By Dr. GEO. K. AMERMAN, one of the attending Surgeons.

CASE I.—COMPOUND FRACTURE OF THE LEG.

Michael Forrester, aged 23, born in Ireland, been in this country four years, by occupation a laborer, of moderately temperate habits, and general good health. He was admitted into the hospital July 10th. On the morning of the 10th, while he was engaged at his work on the street, a part of an old building undergoing repairs, fell some twenty feet, and struck him a little below the middle of the left leg. The blow was a severe one. The soft parts were extensively contused, both bones broken, and the upper end of the lower fragment of the tibia projected about an inch, through a small valvular wound of the parts overlying it, in such a way as to force the integument before it, and prevent an easy replacement. The hemorrhage was slight, though some of the branches of the anterior tibial artery were evidently lacerated. The fracture was reduced, the limb placed in a fracture box, and cold water dressings constantly applied. An anodyne given at night, to procure sleep. The next morning the inflammation was very moderate, with scarcely any pain or constitutional disturbance.

*July 27th.*—External wound entirely healed. No attempt at unions. Side splints substituted for the fracture box, and patient allowed to be up about the ward.

*August 20th.*—No union.

*Sept. 1st.*—Tibia just beginning to unite, though freely moved, and unable to bear the weight of the foot.

*Sept. 10th.*—Union more firm, but very imperfect.

*Sept. 16th.*—This patient is still an inmate of the Hospital. He has been under our charge nearly two and a half months, and promises to remain, at least, another half month. The slow process of union we can trace to no cause, general or local.

CASE II.—SEVERE RAILROAD INJURY OF THE LEG.—AMPUTATION.—DEATH.

Ole Johnson, aged 45, born in Norway, a laborer, of intemperate habits, but general health, good. He was admitted into the Hospital on Monday, July 27th, late in the afternoon. At the time of his admission he was emerging from a state of deep intoxication. About two hours previous to his admission, as he was crossing the railroad track, a train of heavily loaded cars ran over or against him, in such a way as to fracture both bones of the leg, the fibula in several places, open extensively the ankle joint, lacerate all the soft parts on the outside of the lower half of the leg, and cut off, apparently, the supply of blood to the foot, no pulsation could be felt in either the anterior or posterior tibial arteries. The foot was cold, and toes blanched. He complained of no pain, except of the thigh of the opposite side which was badly contused. The right shoulder was, also, severely bruised. No injury about the head or chest perceived on admission. The wound of the soft parts of the leg, and about the ankle, which must have, necessarily from its extent and position, lacerated the posterior tibial artery, still bled very sparingly. Drs. Miller, Duck and Powel were consulted, and amputation of the leg, just below the knee, was thought to offer him his only chance of a recovery. The age of the patient, his intemperate habits, the extent of the injury, its involving so much of the ankle joint, and in all probability cutting off entirely all supply of blood to the foot, were the principal arguments in favor of the operation. The patient readily assented, and 8 o'clock, P. M., was the time fixed for operating.

*July 27th, 8 o'clock, P. M.*—Reaction fully established. Pulse good. Considerable pain in the leg and right shoulder. The patient was anaesthised by a mixture of Chloroform and Ether, (Chloroform 1 part, Ether 5,) and kept so throughout the operation. Very small quantity of blood lost, pulse remained good. Dressed stump with sutures and adhesive straps over it, lint, kept wet in cold water. Gave a full anodyne to procure sleep.

*July 28th.*—Patient been vomiting almost incessantly all night. Pulse accelerated and weakened; surface cool and moist; stump in good condition. No pain. Nausea and vomiting continues.

Ordered Tincture Nux Vomica, five drops, every hour for the vomiting, light nourishing diet during the day, cold water dressings to the stump, and an anodyne at night.

*July 29th.*—Vomiting stopped after second dose of Nux Vomica. Feels well; no pain in stump; slept about six hours last night; stump dressed for the first time; looking well; a piece of lint, covering the end of the stump, and bandage were applied, the lint having been first wet in cold water. The constant application of water discontinued.

*July 31st.*—Everything favorable; dressed the stump, and ordered Quinine in small doses.

*August 2d.*—General condition good. Stump in good condition; dressed daily since last note, and wound united throughout nearly its whole extent; discharge healthy. Observed today, for the first, a soft, boggy swelling about the right elbow joint, also, some pain on moving the joint. Pulse good.

Ordered more generous diet, and increased the Quinine to 5 grains *ter die.*

*August 5th.*—Pulse 140, almost imperceptible; skin cool; countenance haggard; elbow remains swollen. No pain. Discharge from stump scanty and offensive.

Ordered Brandy, Beef Tea and Quinine; two former, ad. lib., the latter, 5 grs. every three hours.

*August 7th.*—Somewhat better; swelling at elbow nearly subsided; pulse 100; skin warm and moist; countenance more calm. Stump still secretes a small amount and unhealthy. Continue Treatment.

*August 8th.*—Called to my patient early this morning, and found him much worse. Pulse 140; very weak; resp. 60; skin cool; *stump dry;* severe pain in chest; no cough; no expectoration.

Ordered Dry Cups to chest, fol. by blister. Stimulants and Quinine as before. He grew rapidly worse and died at 6 o'clock, P. M., from exhaustion and dyspnoea.

The cause of death in the above case, I believe to have been pyemia. The operation was performed July 27th, and every thing promised well until August 2d—six days—when we observed a different swelling about the elbow joint, some pain, some general uneasiness, and anorrexia. These were the first tokens of trouble. Two days afterwards, August 5th, we find our patient much worse; his countenance haggard, pulse rapid and weak, skin cool and moist, elbow swollen, discharge from the wound scanty and offensive. These symptoms are relieved for a time, by stimulants and Quinine; but unfortunately, three days afterwards, chest symptoms supervene, and the patient speedily succumbs.

Pyemia is one of the most fatal consequences that can happen after surgical operations. Its fatality and its frequency, make it of the first importance to the surgeon, and we propose to study it more fully at some future time.

### CASE III.—SIMPLE FRACTURE OF TIBIA.

Michael Leahy, aged 30, born in Ireland, laborer, temperate habits, and good constitution. Admitted into the Hospital August 1st. He received his injury about two hours previous to his admission, by the falling of a heavy timber which he was helping remove. It struck him just above the Int. Malleolus, and produced a transverse fracture of the lower end of the Tibia. The soft parts were very slightly contused. The limb was placed in a fracture box, and cold water dressings used for six days. Side splints were then applied and left for twentytwo days, at the end of which time, union was firm and the patient discharged, cured.

CASE IV.—SEVERAL SEVERE SCALP WOUNDS WITH FRACTURE OF THE SCULL.

Peter Higgins, aged 27, born in Ireland, laborer, of intemperate habits, but general health good. On Saturday night, Sept. 12th, he was attacked and severely beaten by two ruffians, whose intent, it is supposed, was to murder him. The first blow was given by a large heavy club, which knocked him down. When down, they struck him a number of times, but, as he was insensible, it is impossible to ascertain how many. He was found in this condition by a policeman, and taken to the Station House. Two physicians were summoned and carefully dressed his wounds. The next day, Thursday, Sept. 13th, he was sent to the Hospital and placed in my charge. There were six scalp wounds, principally on the right side. The largest was about six inches in length, and extended down to the bone. Another crossed it at right angles. Two were over the mastoid process of the temporal bone, and two in front of the ear. I did not examine for fracture of the scull, as the wounds had been dressed previous to admission. He was also bruised about the face and neck. The physician who dressed his wounds told me his scull was broken. There were no symptoms of compression. Cold water dressings were kept constantly applied, and the next morning he was every way comfortable.

Ordered a mild cathartic, and continued the cold water dressings.

*Sept. 19th.*—Patient has been under our charge one week. There has not been a single unfavorable or suspicious symptom. The wounds are all well, with the exception of the longest. Appetite good. Sleeps well at night, and feels as well as ever. He leaves the Hospital for Kenosha, his place of residence, to-day.

We would be unwilling to say to the above patient that he stands no chance of future trouble. It is well known that secondary accidents not unfrequently occur a long time after injuries of the scalp, and no one could be certain of an immunity, even though he had passed a month without an unfavorable sign.

